# (*E*)-1-(4-Bromo­phen­yl)-2-(4-*tert*-butyl­phen­yl)-1-phenyl­ethene

**DOI:** 10.1107/S1600536808000998

**Published:** 2008-01-16

**Authors:** Chul-Bae Kim, Chul-Hee Cho, Kyu Yun Chai, Kwangyong Park

**Affiliations:** a School of Chemical Engineering and Materials Science, Chung-Ang University, Seoul 156-756, South Korea; b Department of Bionanochemistry, Wonkwang University, Iksan, Chonbuk 570-749, South Korea

## Abstract

In the structure of the title compound, C_24_H_23_Br, the configuration about the double bond is *E*. The dihedral angles between the *tert*-butyl-substituted benzene ring and the unsubstituted and Br-substituted rings are 57.1 (2) and 78.2 (2)°, respectively. The methyl groups are disordered over two positions; the site occupancy factors are *ca* 0.8 and 0.2.

## Related literature

For background, see: Mooney *et al.* (1984 ▶), Kraft *et al.* (1998 ▶), and Martin & Diederich (1999 ▶). For related structures, see: Gao *et al.* (2006 ▶), De Borger *et al.* (2005 ▶), Ogawa *et al.* (1992 ▶); Barnes & Chudek (2002 ▶); SethuSankar *et al.* (2003 ▶).
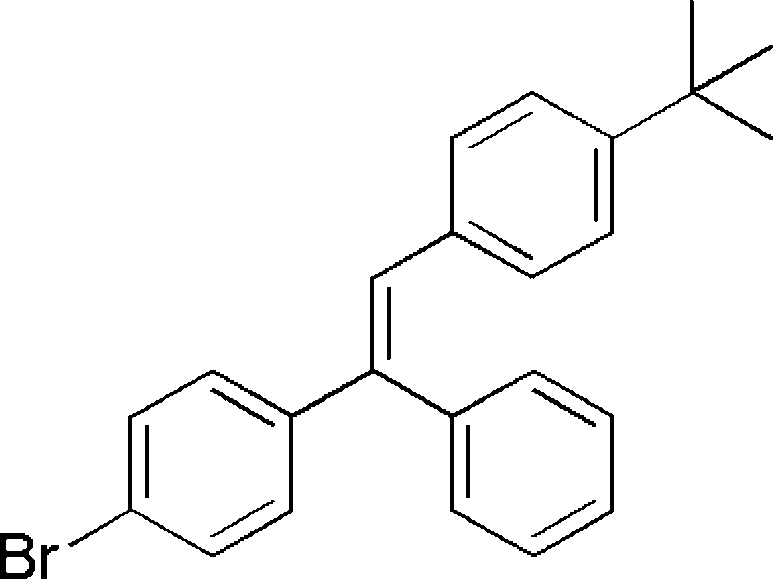


## Experimental

### 

#### Crystal data

C_24_H_23_Br
*M* *_r_* = 391.33
Triclinic, 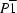

*a* = 8.2950 (4) Å
*b* = 10.8173 (5) Å
*c* = 13.0447 (6) Å
α = 112.415 (3)°
β = 94.570 (3)°
γ = 105.402 (3)°
*V* = 1021.47 (8) Å^3^
*Z* = 2
Mo *K*α radiation
μ = 2.02 mm^−1^
*T* = 296 (2) K
0.45 × 0.40 × 0.04 mm

#### Data collection

Bruker SMART CCD area-detector diffractometer
Absorption correction: multi-scan (*SADABS*; Sheldrick, 1996 ▶) *T*_min_ = 0.413, *T*_max_ = 0.923
17917 measured reflections
4046 independent reflections
2070 reflections with *I* > 2σ(*I*)
*R*_int_ = 0.052

#### Refinement

*R*[*F* ^2^ > 2σ(*F* ^2^)] = 0.051
*wR*(*F* ^2^) = 0.177
*S* = 1.03
4046 reflections
261 parameters
9 restraints
H-atom parameters constrained
Δρ_max_ = 0.47 e Å^−3^
Δρ_min_ = −0.40 e Å^−3^

### 

Data collection: *SMART* (Bruker, 1998 ▶); cell refinement: *SAINT* (Bruker, 1998 ▶); data reduction: *SAINT*; program(s) used to solve structure: *SHELXS97* (Sheldrick, 2008 ▶); program(s) used to refine structure: *SHELXL97* (Sheldrick, 2008 ▶); molecular graphics: *ORTEP-3* (Farrugia, 1997 ▶); software used to prepare material for publication: *SHELXTL* (Sheldrick, 2008 ▶).

## Supplementary Material

Crystal structure: contains datablocks global, I. DOI: 10.1107/S1600536808000998/tk2234sup1.cif

Structure factors: contains datablocks I. DOI: 10.1107/S1600536808000998/tk2234Isup2.hkl

Additional supplementary materials:  crystallographic information; 3D view; checkCIF report

## Figures and Tables

**Fig. 1.**
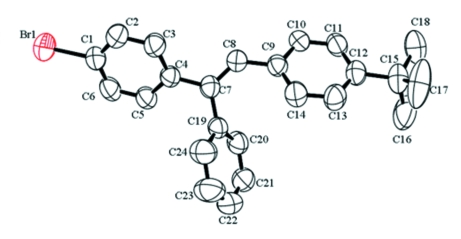
The molecular structure of (I), showing 50% probability displacement ellipsoids and the atom-numbering scheme. For reasons of clarity, the H atoms have been omitted and only the major component of the disordered tert-butyl residue is shown.

**Fig. 2.**
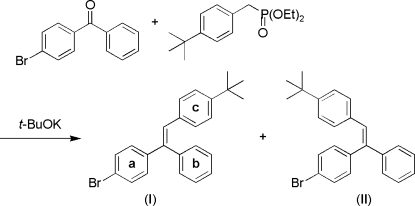
Reaction scheme.

## supplementary crystallographic information

### Comment

Stilbene derivatives are attracting much attention due to their photoluminescent properties (Mooney *et al.*, 1984). Stilbenes, short sub-units of PPV compounds that are important as the emissive layer of organic light-emitting diodes (Kraft *et al.*, 1998), have also been of wide interest in research to gain an understanding of the light emission mechanisms and to predict the influence of substitution on the emitted light (Martin & Diederich, 1999). The title compound (I) was prepared as an important intermediate for such studies by the reaction of 4-bromobenzophenone and diethyl 4-methylbenzylphosphonate using potassium *tert*-butoxide as the base. The reaction produced a 60:40 mixture of (*Z*)- and (*E*)- stereoisomers (Scheme 1). The initial recrystallization process from 2-propanol solution allowed the selective crystallization of the (*Z*)-isomer (II). The title isomer (I) was obtained by consecutive recrystallizations of the remaining mixture from 2-propanol.

The molecular structure of compound (I), Fig. 1, shows that a non-planar conformation is adopted. The torsion angles C7–C8–C9–C10, C8–C7–C4–C5, and C8–C7–C19–C24 are 147.6 (4), 143.4 (4), and 123.5 (5)°, respectively. The dihedral angles between ring a and ring c, and ring b and ring c are 78.2 (2) and 57.1 (2)°, respectively. Steric hindrance between ring b and ring c creates a significant distortion around the ethylene group to widen the angle C7–C8–C9 to 129.2 (3)°. The bond length of C7=C8 is 1.347 (5) Å, which is at the longer end of such bonds reported in *trans*-stilbenes (Gao *et al.*, 2006; De Borger *et al.*, 2005; Ogawa *et al.*, 1992) and in *cis*-stillbenes (Barnes & Chudek, 2002; SethuSankar *et al.*, 2003).

### Experimental

To the mixture of 4-*tert*-butylbenzyl phosphonate (35.1 mmol, 9.98 g) and potassium *tert*-butoxide (54.0 mmol, 6.06 g) in THF (54 ml) was added a solution of 4-bromobenzophenone (27.0 mmol, 5.00 g) in THF (200 ml) at room temperature under an Ar atmosphere. The mixture was stired at refluxing temperature for 3 h. The reaction mixture was cooled to room temperature, diluted with ethyl acetate (300 ml), washed with 1% aqueous HCl (200 ml), water (300 ml) and brine, dried over MgSO_4_, and concentrated *in vacuo*. The crude product, which was a 60:40 mixture of (*Z*)- and (*E*)-stereoisomers by GC analysis, was initially recrystallized from 2-propanol solution to generate the (*Z*)-isomer (II) as pure crystals. The title isomer (I) was obtained by the consecutive recrystallization of the remaining mixture from 2-propanol solution.

### Refinement

The H atoms were included in their idealized positions and refined riding on the corresponding C atoms with C—H = 0.93 to 0.96 Å, and with *U*_iso_ = 1.2*U*_eq_(C) or 1.5*U*_eq_(C) for methyl-H. The methyl groups of the *tert*-butyl residue were found to be disordered over two positions and from refinement, the major component had a site occupancy factor = 0.791 (17).

### Crystal data

**Table:**

C_24_H_23_Br	*Z* = 2
*M**_r_* = 391.33	*F*_000_ = 404
Triclinic, *P*1	*D*_x_ = 1.272 Mg m^−^^3^
Hall symbol: -P 1	Mo *K*α radiation λ = 0.71073 Å
*a* = 8.2950 (4) Å	Cell parameters from 17917 reflections
*b* = 10.8173 (5) Å	θ = 1.7–26.2º
*c* = 13.0447 (6) Å	µ = 2.02 mm^−^^1^
α = 112.415 (3)º	*T* = 296 (2) K
β = 94.570 (3)º	Plate, colourless
γ = 105.402 (3)º	0.45 × 0.40 × 0.04 mm
*V* = 1021.47 (8) Å^3^	

### Data collection

**Table:**

Bruker SMART CCD area-detector diffractometer	4046 independent reflections
Radiation source: fine-focus sealed tube	2070 reflections with *I* > 2σ(*I*)
Monochromator: graphite	*R*_int_ = 0.052
*T* = 293(2) K	θ_max_ = 26.2º
ω scans	θ_min_ = 1.7º
Absorption correction: multi-scan(SADABS; Sheldrick, 1996)	*h* = −10→10
*T*_min_ = 0.413, *T*_max_ = 0.923	*k* = −13→13
17917 measured reflections	*l* = −16→16

### Refinement

**Table:**

Refinement on *F*^2^	Secondary atom site location: difference Fourier map
Least-squares matrix: full	Hydrogen site location: inferred from neighbouring sites
*R*[*F*^2^ > 2σ(*F*^2^)] = 0.051	H-atom parameters constrained
*wR*(*F*^2^) = 0.177	*w* = 1/[σ^2^(*F*_o_^2^) + (0.0939*P*)^2^ + 0.1263*P*] where *P* = (*F*_o_^2^ + 2*F*_c_^2^)/3
*S* = 1.03	(Δ/σ)_max_ = 0.019
4046 reflections	Δρ_max_ = 0.47 e Å^−^^3^
261 parameters	Δρ_min_ = −0.40 e Å^−^^3^
9 restraints	Extinction correction: SHELXL97 (Sheldrick, 1997), Fc^*^=kFc[1+0.001xFc^2^λ^3^/sin(2θ)]^-1/4^
Primary atom site location: structure-invariant direct methods	Extinction coefficient: 0.025 (6)

### Special details

**Table:**

Geometry. All e.s.d.'s (except the e.s.d. in the dihedral angle between two l.s. planes) are estimated using the full covariance matrix. The cell e.s.d.'s are taken into account individually in the estimation of e.s.d.'s in distances, angles and torsion angles; correlations between e.s.d.'s in cell parameters are only used when they are defined by crystal symmetry. An approximate (isotropic) treatment of cell e.s.d.'s is used for estimating e.s.d.'s involving l.s. planes.
Refinement. Refinement of *F*^2^ against ALL reflections. The weighted *R*-factor *wR* and goodness of fit *S* are based on *F*^2^, conventional *R*-factors *R* are based on *F*, with *F* set to zero for negative *F*^2^. The threshold expression of *F*^2^ > 2σ(*F*^2^) is used only for calculating *R*-factors(gt) *etc*. and is not relevant to the choice of reflections for refinement. *R*-factors based on *F*^2^ are statistically about twice as large as those based on *F*, and *R*- factors based on ALL data will be even larger.

### Fractional atomic coordinates and isotropic or equivalent isotropic displacement parameters (Å^2^)

**Table:**

	*x*	*y*	*z*	*U*_iso_*/*U*_eq_	Occ. (<1)
Br1	−0.32899 (6)	−0.30686 (6)	0.89730 (5)	0.1269 (4)	
C1	−0.1190 (5)	−0.2364 (4)	0.8586 (4)	0.0756 (11)	
C2	0.0276 (6)	−0.1682 (5)	0.9382 (4)	0.0869 (12)	
H2	0.0260	−0.1546	1.0129	0.104*	
C3	0.1792 (5)	−0.1190 (4)	0.9077 (3)	0.0774 (11)	
H3	0.2794	−0.0724	0.9626	0.093*	
C4	0.1849 (4)	−0.1378 (3)	0.7971 (3)	0.0602 (9)	
C5	0.0313 (5)	−0.2095 (4)	0.7184 (3)	0.0714 (10)	
H5	0.0311	−0.2254	0.6431	0.086*	
C6	−0.1190 (5)	−0.2572 (4)	0.7485 (4)	0.0769 (11)	
H6	−0.2203	−0.3033	0.6946	0.092*	
C7	0.3451 (4)	−0.0855 (4)	0.7616 (3)	0.0605 (9)	
C8	0.4655 (4)	0.0381 (4)	0.8294 (3)	0.0653 (9)	
H8	0.4399	0.0874	0.8985	0.078*	
C9	0.6311 (4)	0.1058 (4)	0.8099 (3)	0.0609 (9)	
C10	0.6965 (5)	0.2522 (4)	0.8553 (3)	0.0676 (10)	
H10	0.6358	0.3055	0.8994	0.081*	
C11	0.8495 (5)	0.3203 (4)	0.8364 (3)	0.0719 (10)	
H11	0.8878	0.4184	0.8671	0.086*	
C12	0.9472 (5)	0.2481 (4)	0.7737 (3)	0.0684 (10)	
C13	0.8849 (5)	0.1029 (4)	0.7326 (4)	0.0791 (11)	
H13	0.9493	0.0503	0.6921	0.095*	
C14	0.7309 (5)	0.0323 (4)	0.7489 (4)	0.0743 (10)	
H14	0.6937	−0.0658	0.7186	0.089*	
C15	1.1138 (5)	0.3218 (5)	0.7477 (4)	0.0879 (13)	
C16	1.0931 (12)	0.2913 (18)	0.6235 (8)	0.153 (6)	0.791 (17)
H16A	0.9873	0.3013	0.5983	0.230*	0.791 (17)
H16B	1.0926	0.1966	0.5808	0.230*	0.791 (17)
H16C	1.1863	0.3565	0.6124	0.230*	0.791 (17)
C17	1.2556 (9)	0.2679 (14)	0.7758 (13)	0.161 (6)	0.791 (17)
H17A	1.2267	0.1691	0.7280	0.242*	0.791 (17)
H17B	1.2689	0.2822	0.8537	0.242*	0.791 (17)
H17C	1.3606	0.3186	0.7634	0.242*	0.791 (17)
C18	1.1673 (13)	0.4847 (8)	0.8113 (11)	0.134 (4)	0.791 (17)
H18A	1.2715	0.5267	0.7926	0.201*	0.791 (17)
H18B	1.1845	0.5112	0.8914	0.201*	0.791 (17)
H18C	1.0791	0.5171	0.7891	0.201*	0.791 (17)
C16A	1.189 (6)	0.217 (4)	0.670 (4)	0.163 (17)	0.209 (17)
H16D	1.2495	0.2567	0.6245	0.245*	0.209 (17)
H16E	1.0985	0.1317	0.6219	0.245*	0.209 (17)
H16F	1.2659	0.1957	0.7148	0.245*	0.209 (17)
C17A	1.236 (4)	0.408 (6)	0.862 (2)	0.21 (4)	0.209 (17)
H17D	1.3447	0.3928	0.8562	0.308*	0.209 (17)
H17E	1.1902	0.3783	0.9175	0.308*	0.209 (17)
H17F	1.2505	0.5061	0.8849	0.308*	0.209 (17)
C18A	1.071 (4)	0.400 (5)	0.684 (5)	0.17 (2)	0.209 (17)
H18D	1.1707	0.4393	0.6594	0.257*	0.209 (17)
H18E	1.0335	0.4746	0.7317	0.257*	0.209 (17)
H18F	0.9820	0.3367	0.6190	0.257*	0.209 (17)
C19	0.3588 (4)	−0.1758 (4)	0.6450 (3)	0.0607 (9)	
C20	0.3716 (5)	−0.1263 (4)	0.5612 (3)	0.0713 (10)	
H20	0.3756	−0.0338	0.5782	0.086*	
C21	0.3785 (6)	−0.2125 (5)	0.4531 (4)	0.0878 (13)	
H21	0.3884	−0.1777	0.3979	0.105*	
C22	0.3709 (7)	−0.3473 (6)	0.4275 (4)	0.1020 (16)	
H22	0.3753	−0.4057	0.3545	0.122*	
C23	0.3567 (8)	−0.3979 (5)	0.5079 (5)	0.1124 (17)	
H23	0.3514	−0.4910	0.4897	0.135*	
C24	0.3503 (6)	−0.3132 (4)	0.6159 (4)	0.0904 (13)	
H24	0.3399	−0.3497	0.6700	0.108*	

### Atomic displacement parameters (Å^2^)

**Table:**

	*U*^11^	*U*^22^	*U*^33^	*U*^12^	*U*^13^	*U*^23^
Br1	0.0816 (4)	0.1320 (6)	0.1210 (5)	−0.0039 (3)	0.0475 (3)	0.0255 (4)
C1	0.059 (2)	0.070 (2)	0.082 (3)	0.0080 (19)	0.020 (2)	0.024 (2)
C2	0.081 (3)	0.097 (3)	0.070 (2)	0.007 (2)	0.023 (2)	0.034 (2)
C3	0.063 (2)	0.091 (3)	0.065 (2)	0.006 (2)	0.0081 (18)	0.032 (2)
C4	0.060 (2)	0.061 (2)	0.056 (2)	0.0114 (17)	0.0101 (17)	0.0268 (17)
C5	0.066 (2)	0.073 (2)	0.061 (2)	0.0054 (19)	0.0054 (18)	0.0265 (19)
C6	0.057 (2)	0.073 (2)	0.081 (3)	0.0045 (18)	0.0010 (19)	0.026 (2)
C7	0.057 (2)	0.063 (2)	0.064 (2)	0.0144 (17)	0.0118 (17)	0.0323 (18)
C8	0.059 (2)	0.068 (2)	0.063 (2)	0.0140 (18)	0.0144 (17)	0.0253 (18)
C9	0.0543 (19)	0.066 (2)	0.058 (2)	0.0129 (17)	0.0073 (16)	0.0268 (17)
C10	0.058 (2)	0.066 (2)	0.063 (2)	0.0114 (17)	0.0095 (17)	0.0165 (18)
C11	0.062 (2)	0.063 (2)	0.079 (2)	0.0093 (18)	0.0089 (19)	0.0255 (19)
C12	0.0526 (19)	0.080 (3)	0.075 (2)	0.0156 (19)	0.0096 (18)	0.039 (2)
C13	0.062 (2)	0.082 (3)	0.099 (3)	0.029 (2)	0.023 (2)	0.038 (2)
C14	0.065 (2)	0.066 (2)	0.094 (3)	0.0215 (19)	0.017 (2)	0.035 (2)
C15	0.057 (2)	0.104 (3)	0.109 (4)	0.013 (2)	0.017 (2)	0.060 (3)
C16	0.095 (6)	0.243 (14)	0.105 (6)	−0.001 (7)	0.034 (4)	0.089 (8)
C17	0.068 (4)	0.245 (13)	0.267 (17)	0.064 (6)	0.062 (7)	0.193 (13)
C18	0.096 (6)	0.115 (6)	0.161 (10)	−0.018 (5)	0.037 (6)	0.059 (6)
C16A	0.160 (19)	0.162 (18)	0.173 (19)	0.043 (10)	0.041 (11)	0.080 (11)
C17A	0.060 (18)	0.26 (7)	0.11 (2)	−0.07 (3)	0.030 (17)	−0.02 (3)
C18A	0.13 (3)	0.17 (4)	0.24 (6)	0.01 (3)	0.12 (4)	0.12 (5)
C19	0.062 (2)	0.057 (2)	0.057 (2)	0.0087 (16)	0.0112 (16)	0.0257 (17)
C20	0.076 (2)	0.064 (2)	0.070 (3)	0.0104 (18)	0.0132 (19)	0.033 (2)
C21	0.096 (3)	0.091 (3)	0.065 (3)	0.007 (2)	0.020 (2)	0.036 (2)
C22	0.119 (4)	0.086 (4)	0.076 (3)	0.015 (3)	0.036 (3)	0.016 (3)
C23	0.165 (5)	0.072 (3)	0.095 (4)	0.038 (3)	0.044 (3)	0.027 (3)
C24	0.126 (4)	0.067 (3)	0.080 (3)	0.024 (2)	0.027 (3)	0.036 (2)

### Geometric parameters (Å, °)

**Table:**

Br1—C1	1.897 (4)	C15—C17A	1.52 (3)
C1—C2	1.356 (6)	C15—C18	1.550 (10)
C1—C6	1.366 (6)	C16—H16A	0.9600
C2—C3	1.381 (5)	C16—H16B	0.9600
C2—H2	0.9300	C16—H16C	0.9600
C3—C4	1.385 (5)	C17—H17A	0.9600
C3—H3	0.9300	C17—H17B	0.9600
C4—C5	1.392 (5)	C17—H17C	0.9600
C4—C7	1.483 (5)	C18—H18A	0.9600
C5—C6	1.366 (5)	C18—H18B	0.9600
C5—H5	0.9300	C18—H18C	0.9600
C6—H6	0.9300	C16A—H16D	0.9600
C7—C8	1.347 (5)	C16A—H16E	0.9600
C7—C19	1.494 (5)	C16A—H16F	0.9600
C8—C9	1.467 (5)	C17A—H17D	0.9600
C8—H8	0.9300	C17A—H17E	0.9600
C9—C14	1.387 (5)	C17A—H17F	0.9600
C9—C10	1.389 (5)	C18A—H18D	0.9600
C10—C11	1.381 (5)	C18A—H18E	0.9600
C10—H10	0.9300	C18A—H18F	0.9600
C11—C12	1.375 (6)	C19—C24	1.366 (5)
C11—H11	0.9300	C19—C20	1.389 (5)
C12—C13	1.380 (5)	C20—C21	1.378 (6)
C12—C15	1.535 (6)	C20—H20	0.9300
C13—C14	1.382 (5)	C21—C22	1.349 (6)
C13—H13	0.9300	C21—H21	0.9300
C14—H14	0.9300	C22—C23	1.356 (7)
C15—C18A	1.48 (4)	C22—H22	0.9300
C15—C16	1.510 (9)	C23—C24	1.372 (6)
C15—C16A	1.52 (3)	C23—H23	0.9300
C15—C17	1.522 (8)	C24—H24	0.9300
			
C2—C1—C6	120.9 (4)	C16A—C15—C12	112.0 (15)
C2—C1—Br1	120.7 (3)	C17—C15—C12	110.2 (4)
C6—C1—Br1	118.4 (3)	C17A—C15—C12	105.1 (12)
C1—C2—C3	119.6 (4)	C18A—C15—C18	63 (2)
C1—C2—H2	120.2	C16—C15—C18	105.4 (7)
C3—C2—H2	120.2	C16A—C15—C18	135.9 (16)
C2—C3—C4	121.3 (4)	C17—C15—C18	110.7 (6)
C2—C3—H3	119.4	C17A—C15—C18	56 (2)
C4—C3—H3	119.4	C12—C15—C18	112.1 (5)
C3—C4—C5	116.9 (3)	C15—C16—H16A	109.5
C3—C4—C7	122.8 (3)	C15—C16—H16B	109.5
C5—C4—C7	120.4 (3)	C15—C16—H16C	109.5
C6—C5—C4	121.9 (4)	C15—C17—H17A	109.5
C6—C5—H5	119.0	C15—C17—H17B	109.5
C4—C5—H5	119.0	C15—C17—H17C	109.5
C1—C6—C5	119.3 (3)	C15—C18—H18A	109.5
C1—C6—H6	120.3	C15—C18—H18B	109.5
C5—C6—H6	120.3	C15—C18—H18C	109.5
C8—C7—C4	121.3 (3)	C15—C16A—H16D	109.5
C8—C7—C19	123.2 (3)	C15—C16A—H16E	109.5
C4—C7—C19	115.4 (3)	H16D—C16A—H16E	109.5
C7—C8—C9	129.2 (3)	C15—C16A—H16F	109.5
C7—C8—H8	115.4	H16D—C16A—H16F	109.5
C9—C8—H8	115.4	H16E—C16A—H16F	109.5
C14—C9—C10	116.5 (3)	C15—C17A—H17D	109.4
C14—C9—C8	123.9 (3)	C15—C17A—H17E	109.5
C10—C9—C8	119.6 (3)	H17D—C17A—H17E	109.5
C11—C10—C9	121.5 (4)	C15—C17A—H17F	109.5
C11—C10—H10	119.2	H17D—C17A—H17F	109.5
C9—C10—H10	119.2	H17E—C17A—H17F	109.5
C12—C11—C10	122.3 (4)	C15—C18A—H18D	109.5
C12—C11—H11	118.8	C15—C18A—H18E	109.4
C10—C11—H11	118.8	H18D—C18A—H18E	109.5
C11—C12—C13	115.9 (3)	C15—C18A—H18F	109.5
C11—C12—C15	123.0 (4)	H18D—C18A—H18F	109.5
C13—C12—C15	121.1 (4)	H18E—C18A—H18F	109.5
C12—C13—C14	122.8 (4)	C24—C19—C20	117.7 (4)
C12—C13—H13	118.6	C24—C19—C7	120.6 (3)
C14—C13—H13	118.6	C20—C19—C7	121.6 (3)
C13—C14—C9	120.9 (4)	C21—C20—C19	120.9 (4)
C13—C14—H14	119.5	C21—C20—H20	119.5
C9—C14—H14	119.5	C19—C20—H20	119.5
C18A—C15—C16	48 (2)	C22—C21—C20	119.9 (4)
C18A—C15—C16A	106 (2)	C22—C21—H21	120.1
C16—C15—C16A	59.9 (18)	C20—C21—H21	120.1
C18A—C15—C17	141.0 (12)	C21—C22—C23	120.1 (4)
C16—C15—C17	107.3 (7)	C21—C22—H22	120.0
C16A—C15—C17	50.0 (19)	C23—C22—H22	120.0
C18A—C15—C17A	117 (2)	C22—C23—C24	120.7 (5)
C16—C15—C17A	143.7 (14)	C22—C23—H23	119.7
C16A—C15—C17A	109 (2)	C24—C23—H23	119.7
C17—C15—C17A	62 (2)	C19—C24—C23	120.8 (4)
C18A—C15—C12	107.4 (11)	C19—C24—H24	119.6
C16—C15—C12	111.0 (4)	C23—C24—H24	119.6
